# Baseline P2Y12 reactivity, kidney function, and *CYP2C19* genotype determine clopidogrel responsiveness in acute stroke

**DOI:** 10.1038/s41598-023-34481-5

**Published:** 2023-05-19

**Authors:** Yi-Chung Lee, Yi-Chu Liao, Chun-Jen Lin, Chih-Ping Chung

**Affiliations:** 1 Department of Neurology, Neurological Institute, Taipei Veterans General Hospital, No. 201, Section 2, Shih-Pai Road, Taipei, 112 Taiwan; 2 Department of Neurology, School of Medicine, National Yang Ming Chao Tung University, Taipei, Taiwan

**Keywords:** Neurology, Stroke

## Abstract

Clopidogrel is the most-widely used platelet P2Y12-inhibitor for secondary-prevention of ischemic stroke. Platelet P2Y12 reactivity before and after inhibitors can be measured with blood sampling by commercialized system. We aimed to evaluate (1) whether high-on-clopidogrel platelet P2Y12 reactivity (HCPR) is associated with short-term vascular events and (2) the predictors of HCPR in acute stroke. The inclusion criterion was patients with acute stroke who received clopidogrel within 12–48 h after the onset. Platelet reactivity was assayed at baseline and after clopidogrel treatment using the VerifyNow system. The primary endpoint was recurrent ischemic events within 21 days after stroke. Among 190 patients, 32(16.9%) had recurrent ischemic stroke. Multivariate analyses showed that HCPR was significantly associated with the short-term events with an odds-ratio of 2.5 (95% CI 1.1–5.7, *p* = 0.027). Patients with HCPR had significantly higher frequencies of high baseline platelet P2Y12 reactivity, impaired kidney function, and carrying one or two *CYP2C19* loss-of-function alleles. A poor clopidogrel response score combining these factors was developed. Ten percent of patients with score 0, 20.3% of those with score 1, 38.3% of those with score 2, and 66.7% of those with score 3 had HCPR (χ^2^-test, *p* < 0.001). Multivariate analyses showed that, compared with the score-0 group, the score-2 and -3 groups had higher risks of HCPR with hazard-ratios of 5.4 (95% CI 1.5–20.3, *p* = 0.012) and 17.4 (95% CI 3.4–88.9, *p* = 0.001) for developing recurrent ischemic strokes. The study emphasized the role of HCPR in ischemic stroke. We also developed an HCPR risk score, which could be used in clinical practice or trials, potentially with more precision, to weigh the clinical benefit of a tailored antiplatelet-strategy for patients with stroke.

## Introduction

Clopidogrel has been increasingly used for stroke prevention at the acute stage of ischemic stroke^1–7^. There is evidence for clopidogrel treatment in the following conditions: (1) short-term (within 21 days after stroke onset) dual antiplatelet therapy (DAPT) with aspirin in patients with minor stroke or high-risk transient ischemic attack (TIA)^1^, (2) isolated clopidogrel in patients with aspirin intolerance^2^ or recurrent stroke after aspirin treatment (aspirin failure cases)^3,4^, and (3) DAPT with aspirin or cilostazol in patients with severe intracranial artery stenosis-related stroke^5,6^. Under these conditions, risks of stroke recurrence are highest at the first few days after stroke onset; therefore, prompt and effective antiplatelet treatment after acute stroke has been encouraged in the stroke guidelines^2,7^.

Clopidogrel reduces adenosine diphosphate-induced platelet aggregation and thrombotic events by inhibiting platelet P2Y12 receptor activity. The pharmacodynamic effects of clopidogrel are subject to significant variability, and a considerable number of patients has inadequate platelet inhibition with persistent high platelet reactivity (high-on clopidogrel platelet reactivity, HCPR)^8,9^. Initially, HCPR was shown to be a predictor of recurrent thrombotic events or stent thrombosis in patients with ischemic heart disease^8–10^. Recent studies on ischemic stroke have also demonstrated a significant, independent association between HCPR and recurrent ischemic events at 3–6 months and even in longer periods of follow-up (up to 54 months)^11–14^. Therefore, it is of clinical importance to evaluate and consider patients with ischemic stroke who are prone to clopidogrel resistance before prescription.

Clopidogrel is a prodrug, requiring metabolism before it can actively decrease the platelet reactivity^8^. Among several metabolic steps, cytochrome P450 2C19 (CYP2C19) is an important enzyme for clopidogrel metabolism^15^. Furthermore, more than half of the Asian population including our own Taiwan population has carried at least one allele of the *CYP2C19* loss-of-function (LoF) variants^16,17^. However, studies to identify patients at risk of HCPR found that *CYP2C19* genotypes explain only a fraction of the pharmacodynamic response of clopidogrel^8,9,18,19^. Meanwhile, studies also found that carriers of *CYP2C19* LoF alleles did not necessarily bring an increased risk of recurrent vascular events^13^. In addition to genetic contributions, a number of clinical factors such as obesity, diabetes mellitus, and chronic kidney disease (CKD) are associated with HCPR^4,9,19,20^. Because previous studies evaluated only the on-clopidogrel platelet P2Y12 reactivity but not baseline P2Y12 reactivity, we wondered whether a high platelet reactivity before clopidogrel usage (baseline P2Y12 reactivity) is associated with HCPR; its potential interactions with other factors associated with HCPR also warrant investigations.

The present study prospectively recruited Taiwanese patients with acute ischemic stroke and clopidogrel prescription within 12–48 h after stroke onset. We measured both baseline and on-clopidogrel (at least after 5 days of usage) platelet P2Y12 reactivity, assessed *CYP2C19* genotypes, and recorded the clinical outcomes at 21 days in all patients. The endpoint observed events included recurrent ischemic stroke or TIA. Through a non-clinical trial of a real-world population, we aimed to evaluate (1) whether HCPR was associated with short-term recurrent ischemic events and (2) the predictors of HCPR at the acute stage of ischemic stroke.

## Methods

### Study population

**Figure 1:**
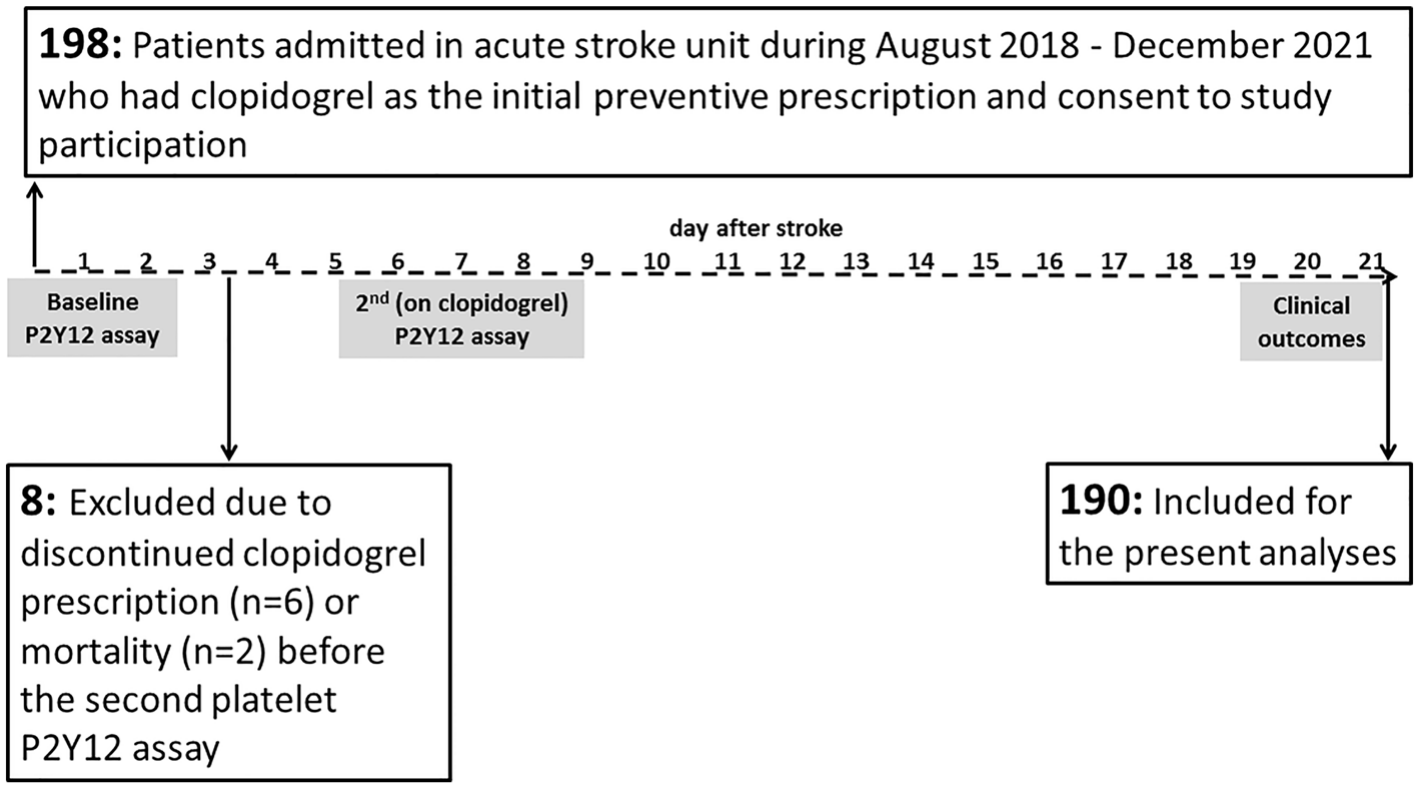
The flowchart of participants’ recruitment and study protocol.

A flowchart of the participant recruitment process and the study protocol is shown in Fig. 1. This was a prospective, observational study. Patients who were admitted to our acute stroke unit (Taipei Veterans General Hospital, Taiwan) between August 2018 and December 2021 were screened for selection. Patients with ischemic stroke, who had clopidogrel prescription within 12–48 h after stroke onset by an in-charge physician and gave consents to participate in the study were eligible for inclusion in the present study. Patients in whom clopidogrel treatment had been prescribed before the present clinical event or was discontinued before the second P2Y12 assay (on-clopidogrel platelet reactivity) were excluded.

### Standard protocol approval and patient consent

This study was approved by the Institutional Review Board of Taipei Veterans General Hospital, Taipei, Taiwan. All participants provided written informed consents. All methods were carried out in accordance with relevant guidelines and regulations.

### Cardiovascular risk factors’ definitions

Cardiovascular risk factors were either measured or assessed by self-report. Presence of hypertension was determined by a self-report of current antihypertensive medication prescription, or by a measurement of either systolic BP ≥ 140 mmHg or diastolic BP ≥ 90 mmHg. Diabetes mellitus was defined by either a self-report of current diabetes medication, or a measurement of hemoglobin A1c (HgbA1c) ≥ 6.5. Body mass index (BMI) was calculated as weight (in kilograms) divided by height (in meters) squared. Obesity was defined as BMI ≥ 30 kg/m^2^. Impaired kidney function was defined by an estimated glomerular filtration rate (eGFR) < 60 mL/min/1.73 m^2^.

### Platelet P2Y12 reactivity assay

The baseline platelet reactivity and on-treatment platelet reactivity were assayed using the VerifyNow System (Accumetrics, San Diego, CA)^13,21^. Baseline platelet P2Y12 reactivity was assessed before or within 24 h after clopidogrel treatment, while the second (on-clopidogrel) platelet P2Y12 reactivity was performed at least 5 days after clopidogrel treatment (Fig. 1). After discarding the first 2 mL of blood, peripheral blood samples from patients’ antecubital veins were collected into 2-mL Vacutainer tubes containing 3.2% sodium citrate. Whole blood was tested within 2 h of collection, as instructed by the manufacturer, using the VerifyNow System. Platelet reactivity was shown in P2Y12 reaction units (PRU), and HCPR was defined as a PRU value of ≥ 208^8,9,13,14^.

### CYP2C19 genotyping

Genomic DNA was isolated from whole blood using the Gentra Puregene kit according to the manufacturer’s protocol (Qiagen, Hilden, Germany). Two single-nucleotide polymorphisms (SNPs) of *CYP2C19* determining the two most frequent LoF alleles CYP2C19*2 (rs4244285, c.681G>A, p.Pro227Pro) and CYP2C19*3 (rs4986893, c.636G>A, p.Trp212Ter) were genotyped in all participants. Genotyping was performed using the TaqMan genotyping assay (Applied Biosystems, Foster City, CA, USA) with SNP probes c_25986767_70 and c_17861809_10 for the CYP2C19*2 allele and the CYP2C19*3 allele, respectively, using an ABI7500 real-time PCR machine (Applied Biosystems). Allele discrimination was achieved by detecting fluorescence using System SDS software, version 1.2.3 (Applied Biosystems). Individuals carrying the AA or AG genotype at rs4244285 were defined as two or one CYP2C19*2 allele carriers, whereas individuals harboring the AA or AG genotype at rs4986893 were defined as two or one CYP2C19*3 allele carriers. We further classified the subjects into three groups according to their putative CYP2C19 activity to convert clopidogrel into active metabolites. Subjects with two *2, one *2 plus one *3 or two *3 alleles (*2/*2, *2/*3, or *3/*3) were classified as poor metabolizers because they had the worst clopidogrel metabolism and activation, while those with only one *2 or *3 allele (were classified as intermediate metabolizers^15,17^.

### Assessment of clinical outcomes

The primary endpoint was a composite of recurrent ischemic stroke or TIA. TIA was defined as focal brain ischemia with resolution of symptoms within 24 h after stroke onset together with a moderate-to-high risk of stroke recurrence (defined as a score of ≥ 4 at the time of randomization on ABCD2). Recurrent ischemic stroke was defined as an acute focal infarction of the brain or retina with one of the following features: (1) sudden onset of a new focal neurologic deficit, with clinical or imaging evidence of infarction lasting for 24 h or more and not attributable to a non-ischemic cause; (2) a new focal neurologic deficit lasting for less than 24 h and not attributable to a non-ischemic cause but accompanied by neuroimaging evidence of new brain infarction; (3) rapid worsening of an existing focal neurologic deficit lasting for more than 24 h and not attributable to a brain hemorrhagic lesion, post-infarct brain edema, or systemic medical cause such as infection (stroke-in-evolution).

### Statistical analyses

Data analyses were performed using SPSS software (version 22.0; IBM, USA). All data are presented as the mean (standard deviation) for continuous variables and as number (percentage) for discrete variables. Group comparisons were made using nonparametric Mann–Whitney *U* test. Chi-square (χ^2^) or Fisher’s exact test was performed for categorical variables. Multivariate analyses were used to determine the relationship between each factor and the occurrence of short-term events or HCPR. Confounding factors such as age, sex, and vascular risk factors were adjusted. Odds ratios (ORs) or hazard ratios (HRs) and 95% confidence intervals (CIs) were calculated. Statistical significance was set at *p* < 0.05.

### Ethical approval and consent to participate

This study was approved by the Institutional Review Board of Taipei Veterans General Hospital, Taipei, Taiwan. All participants provided written informed consents.

## Results

Among 198 eligible patients, six who discontinued clopidogrel treatment within 21 days after stroke onset and two who died before the second P2Y12 reactivity measurement were excluded. Data from the remaining 190 patients were included in the analysis (Fig. 1). Among them, 29 (15.3%) patients had received intravenous alteplase thrombolysis, 24 (12.6%) had received intra-arterial thrombectomy and 6 (3.2%) had received both. All the patients received clopidogrel treatment during the 21-day observational period. Patients who did not receive acute thrombolytic therapy (n = 161) were prescribed clopidogrel within 12–24 h after stroke onset, whereas the other patients were prescribed clopidogrel within 24–48 h after stroke onset (at least 24 h after thrombolytic therapy). One hundred thirteen (59.5%) patients received DAPT with aspirin (100 mg per day), and 33 (17.4%) patients received an initial clopidogrel loading (75 mg × 3 tablets = 225 mg). The clinical and demographic characteristics of the patients are presented in Table 1. Among these participants, 80 (42.1%) were minor stroke patients with National Institutes of Health stroke scale (NIHSS) ≤ 3, and 63 (33.2%) had previous ischemic stroke events.

**Table 1:** Comparison of clinical and demographic characteristics between the groups.

Characteristic	Total	Events within 21 days after stroke		p*
		−	+	
Number of patients	190	158	32	
Age, years, mean (range, SD)	69.5 (29–94, 13.5)	69.3 (13.2)	70.8 (15.1)	0.573
Male sex, n (%)	125 (65.8)	101 (63.9)	24 (75.0)	0.307
Infarct region, n (%)				
Anterior circulation	139 (73.2)	112 (70.9)	27 (84.4)	0.131
Posterior circulation	55 (28.9)	50 (31.6)	5 (15.6)	0.087
Stroke severity, NIHSS, mean (range, SD)	7.4 (0–36, 7.0)	7.6 (7.3)	6.4 (5.6)	0.385
Minor stroke (NIHSS ≤ 3), n (%)	80 (42.1)	66 (41.8	14 (43.8)	0.847
Vascular risk factors, n (%)				
Hypertension	148 (77.9)	119 (75.3)	29 (90.6)	0.064
Diabetes mellitus	67 (35.3)	52 (32.9)	15 (46.9)	0.157
Dyslipidemia	112 (58.9)	92 (58.2)	20 (62.5)	0.698
Cigarette smoking	69 (36.3)	56 (35.4)	13 (40.6)	0.687
Impaired kidney function†	57 (30.0)	43 (27.2)	14 (43.8)	0.089
Obesit^y‡^	14 (7.4)	10 (6.4)	4 (12.9)	0.255
Previous stroke events	63 (33.2)	53 (33.5)	10 (31.3)	0.274
Thrombolytic treatment, n (%)				
Intravenous alteplase thrombolysis	29 (15.3)	26 (16.5)	3 (9.4)	0.423
Intra-arterial thrombectomy	24 (12.6)	22 (13.9)	2 (6.3)	0.380
Dual antiplatelet therapy with clopidogrel plus aspirin, n (%)	113 (59.5)	91 (57.6)	22 (68.8)	0.324
Initial clopidogrel loading, n (%)	33 (17.4)	26 (16.5)	7 (21.9)	0.450
High platelet P2Y12 reactivity (PRU ≥ 208), n (%)				
Baseline	68 (35.8)	58 (36.7)	10 (31.3)	0.430
After clopidogrel treatment	52 (27.4)	38 (24.1)	14 (43.8)	0.029
*CYP2C19* loss-of-function allele carrier, n (%)				
One allele (intermediate metabolizer)	104 (54.7)	86 (54.4)	18 (56.3)	0.953
Two alleles (poor metabolizer)	27 (14.2)	23 (14.6)	4 (12.5)	

*NIHSS* National Institutes of Health stroke scale, *PRU* P2Y12 reaction units.

*Comparisons between patients with and without short-term events within 21 days of acute ischemic stroke. ^†^Estimated glomerular filtration rate (eGFR) < 60 mL/min/1.73 m^2^. ^‡^Body mass index of ≥ 30 kg/m^2^.

### HCPR is associated with short-term events after acute stroke

Within 21 days of stroke onset, 32 (16.9%) patients experienced recurrent ischemic stroke. There was no recurrent TIA. Comparisons between patients with and without events showed similar clinical and demographic characteristics, although there were borderline significantly higher frequencies of hypertension and impaired kidney function in patients with events (Table 1).

Results of analyses to evaluate whether HCPR was associated with short-term events after acute ischemic stroke showed that the event group had a significantly higher frequency of HCPR (45.2% vs. 24.8%, *p* = 0.029) than the non-event group (Table 1). To validate this association, we performed multivariate analyses adjusted for age, sex, and/or vascular risk factors (Table 2). For adjusted vascular risk factors, we chose hypertension and impaired kidney function, which had borderline significant associations with clinical events (Table 1). The results showed that HCPR was significantly and independently associated with short-term events after acute stroke, with an OR of 2.5 (Table 2). We did not find any other factors, including the *CYP2C19* LoF genotypes, associated with the short-term events.

**Table 2:** Multivariate analyses of the associations between high-on-clopidogrel platelet P2Y12 reactivity and short-term events after acute stroke.

	Short-term events after stroke		
	OR	95% CI	*p*
High-on-clopidogrel platelet P2Y12 reactivity			
Model 1: Adjusted for age and sex	2.5	1.1–5.6	0.026
Model 2: Adjusted for age, sex, HTN, and impaired kidney function	2.5	1.1–5.7	0.027

*OR* odds ratio, *CI* confidence interval, *HTN* hypertension.

### Baseline platelet P2Y12 reactivity, kidney function, and CYP2C19 genotype are associated with HCPR

We also assessed whether baseline platelet P2Y12 reactivity or other factors could predict HCPR in the acute stage of stroke. Baseline platelet P2Y12 reactivity results were not associated with the interval time (hour) between the first dose of clopidogrel-taken and assessment time points. Table 3 shows a comparison between patients with and without HCPR. The results showed that patients with HCPR had significantly higher frequencies of high baseline platelet P2Y12 reactivity, impaired kidney function, and being *CYP2C19* LoF allele carriers, particularly poor metabolizers (Table 3). Multivariate analyses further validated these three factors as independent predictors of HCPR (Table 4). Notably, obesity and diabetes mellitus were not associated with HCPR in our study population.

**Table 3:** Comparison between patients with and without high-on-clopidogrel platelet P2Y12 reactivity.

	PRU < 208	PRU ≥ 208	*p*
Number of patients	132	52	
Age, years, mean (SD)	68.6 (13.8)	71.6 (13.0)	0.176
Male sex, n (%)	87 (65.9)	33 (63.5)	0.864
Vascular risk factors, n (%)			
Hypertension	103 (78.0)	42 (80.8)	0.842
Diabetes mellitus	43 (32.6)	20 (38.5)	0.492
Dyslipidemia	77 (58.3)	31 (59.6)	1.000
Cigarette smoking	48 (36.4)	19 (36.5)	1.000
Impaired kidney function	30 (22.7)	23 (44.2)	0.006
Obesity	11 (8.5)	3 (5.8)	0.760
Previous stroke events	40 (30.3)	19 (36.5)	0.756
Dual antiplatelet therapy with clopidogrel plus aspirin, n (%)	73 (55.3)	34 (65.4)	0.247
Initial clopidogrel loading, n (%)	22 (16.7)	11 (21.2)	0.524
High platelet P2Y12 reactivity at baseline, n (%)	38 (28.8)	27 (51.9)	< 0.001
*CYP2C19* loss-of-function allele carrier, n (%)			
One allele (intermediate metabolizer)	68 (51.5)	31 (59.6)	0.034
Two alleles (poor metabolizer)	16 (12.1)	11 (21.2)	

*PRU* P2Y12 reaction unit, *SD* standard deviation.

**Table 4:** Multivariate analyses of the associations between each factor and high-on-clopidogrel platelet P2Y12 reactivity.

Factor	High-on-clopidogrel platelet P2Y12 reactivity		
	OR	95% CI	*p*
Age	1.0	0.9–1.0	0.301
Female sex	1.5	0.6–4.1	0.403
High platelet P2Y12 reactivity at baseline	9.3	2.9–30.4	< 0.001
*CYP2C19* loss-of-function allele carrier	3.5	1.2–10.5	0.026
Impaired kidney function	3.8	1.3–11.1	0.016

*OR* odds ratio, *CI* confidence interval.

### Poor clopidogrel responsive score predicts HCPR at the acute stage of stroke

**Figure 2:**
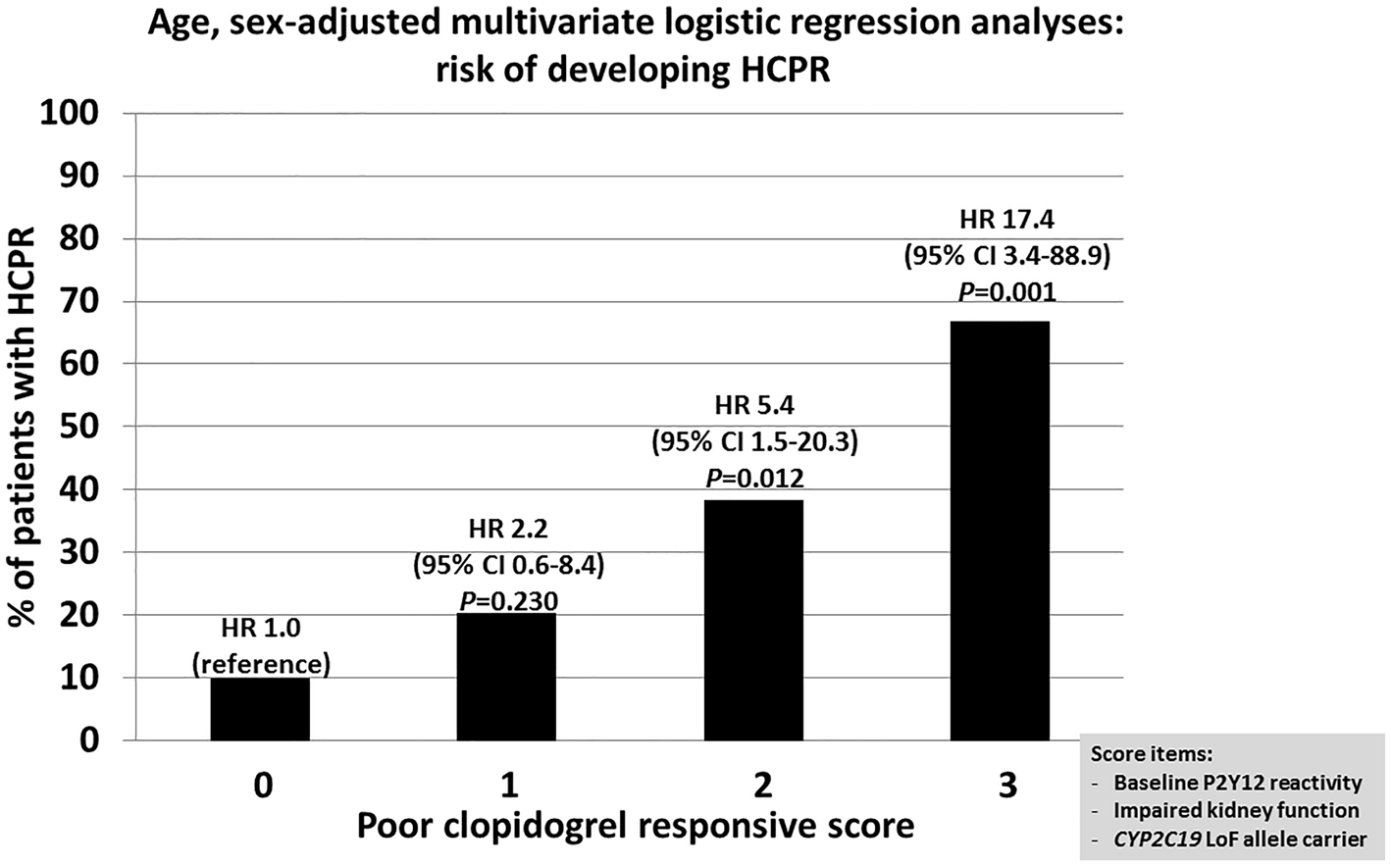
Poor clopidogrel response score predicts high-on-clopidogrel platelet reactivity (HCPR).

We then created a poor clopidogrel response score by combining (1) high baseline platelet P2Y12 reactivity, (2) impaired kidney function, and (3) *CYP2C19* LoF allele carriers. The score ranged from 0 to 3 points with one point added to the score at the presence of each the three above mentioned conditions. Thirty (15.8%), 80 (42.1%), 64 (33.7%), and 16 (8.4%) patients had scores of 0, 1, 2, and 3, respectively. Three (10.0%) patients in the score-0 group, 16 (20.3%) patients in the score-1 group, 23 (38.3%) patients in the score-2 group, and 10 (66.7%) patients in the score-3 group had HCPR (χ^2^ test, *p* < 0.001; Fig. 2). Multivariate logistic regression analyses adjusted for age and sex showed that, compared with the zero score, scores 2 and 3 had significantly higher risks of HCPR, with HR of 5.4 and 17.4, respectively (Fig. 2).

## Discussion

The present study prospectively recruited patients with ischemic stroke and clopidogrel prescription and found that (1) HCPR was associated with short-term (within 21 days) recurrent events, and (2) factors including high platelet P2Y12 reactivity at baseline, impaired kidney function, and *CYP2C19* LoF allele carriers were independently and significantly associated with HCPR. Poor clopidogrel response scores of 2 and 3 when combining these factors had HRs of 5.4 and 17.4 for developing HCPR, respectively.

Large clinical studies have consistently found that most recurrent events occur within a short period (10–21 days) after acute ischemic stroke^1,5^. They also showed that even with prompt anti-thrombotic treatment after acute ischemic stroke, there was a still considerable recurrence event rate, particularly at the acute stage^1,2,5,7^. Clopidogrel is widely used for stroke prevention at the acute stage of ischemic stroke^1–7^. It is clinically urgent to find which factor could predict short-term recurrent events and ways to identify those who are at risk. In the present study, 32 (16.8%) patients developed recurrent ischemic events within 21 days after acute ischemic stroke, even with prompt clopidogrel use. In addition, echoing the findings of other studies that reported the prevalence of HCPR ranging from 21.5 to 55.5%^13^, there was a considerable proportion of patients with HCPR (n = 57, 27.4%) in our stroke patients. The results showed that HCPR was the only factor significantly and independently associated with occurrence of short-term events. This finding is consistent with those of previous studies that high on-medicines platelet reactivity, as evaluated through the direct measurement of the antiplatelet effect of the drug, a powerful and independent parameter predicting both short-term and long-term recurrent vascular events^9–14^. Given the above lines of evidence, anti-thrombotic therapy other than clopidogrel should be considered in patients with stroke and HCPR. However, on-clopidogrel platelet reactivity can be evaluated only after 5–7 days of treatment (waiting for clopidogrel to achieve its steady-state effects), when recurrent ischemic events might have already occurred^1,5^. It is necessary to obtain information indicating HCPR before or at least a few days after antiplatelet agent prescription in patients with acute ischemic stroke. Several studies, therefore, aimed to identify the genetic or clinical factors that would influence platelet response to clopidogrel.

The most common genetic factor described in the literature related to HCPR is the *CYP2C19* LoF genotypes^8–10,13–16,18,19^. In these studies, *CYP2C19* LoF allele carriers had an OR of 2–3 of HCPR. Our study also showed an OR of 3.5 for developing HCPR in patients carrying one or two *CYP2C19* LoF alleles. The prevalence of *CYP2C19* LoF allele carrier is higher in the Asian population than in the western populations (one allele carrier: 60% versus 30%; two alleles carrier: 10% versus 3%)^8–10,13–19^. The prevalence in the present study population (*CYP2C19* LoF one allele carrier: 54.7%, *CYP2C19* LoF two alleles carrier: 14.2%) was similar with our previous study of a larger Taiwanese population^17^. Given that being the strongest effective and safe platelet P2Y12 inhibitor for stroke prevention at the current time and having many lines of evidence supporting its therapeutic benefits, clopidogrel is the most broadly used platelet P2Y12 inhibitor for stroke patients^1–7^. Therefore, the *CYP2C19* genotype might be the most important pharmacogenetic factor in the Asian stroke population.

The most significant clinical factor associated with HCPR in the present study was impaired kidney function, which can be measured soon after hospital arrival in patients with acute stroke. A poorer response to clopidogrel in CKD patients has also been reported in previous studies^8,9,20,22,23^. The mechanisms for the decreased clopidogrel effect in patients with impaired kidney function are not clear yet and might be explained by disturbed bioavailability or metabolism of drugs, relevant peptides, hematological cells, and cytokines, increased platelet turnover rate, or uremic toxins^24^.

Other clinical factors commonly reported to be associated with HCPR are diabetes mellitus^8,9,13^. Conversely, several studies did not show similar findings regarding the association between diabetes mellitus and HCPR^25,26^. This may suggest that the duration and severity of hyperglycemia may modify its influence on the antiplatelet effect of clopidogrel. In addition to diabetes mellitus, obesity has been reported as an HCPR-related factor^8,9,13^, mostly in the western populations, in which obesity is more prevalent than that in the Asian population^27^. We postulate that one of the reasons why our population did not show an association between obesity and HCPR might be related to its low prevalence (7.4%) in our study population of all Asian ethnicities. These results indicate that different algorithms should be developed to predict the risk of HCPR in populations with different characteristics, particularly ethnicity.

This is the first study to report that high baseline platelet P2Y12 reactivity (PRU value of ≥ 208) is a significant determinant of HCPR. Because most studies recruited on-clopidogrel patients beyond the acute stage, baseline platelet reactivity before clopidogrel usage (baseline P2Y12 reactivity) was unknown; therefore, it could not be evaluated in previous studies. In our population, 87.1% of the patients with high baseline platelet P2Y12 reactivity had HCPR. Furthermore, among the three associated clinical factors, high baseline platelet P2Y12 reactivity was associated with the highest risk (OR = 9.3). In our study, 22.7% of patients with impaired kidney function, 51.5% of patients with one *CYP2C19* LoF allele, and 12.1% of patients with two *CYP2C19* LoF alleles had normal on-clopidogrel platelet P2Y12 reactivity. Because the definition of HCPR is a PRU value of ≥ 208 under clopidogrel treatment, it is reasonable to presume that patients with normal baseline platelet P2Y12 reactivity are less likely to develop HCPR even when they have one or two *CYP2C19* LoF alleles or other clinical factors affecting clopidogrel effects. The critical role of baseline platelet reactivity in HCPR also partly explains why *CYP2C19* LoF genotypes and other clinical factors contribute only to a fraction of the pharmacodynamic response to clopidogrel. Patients with higher platelet reactivity at baseline are prone to atherothromboembolic formation and thus might have a higher risk of recurrent ischemic events. Therefore, a prompt and effective antiplatelet strategy is important for these patients. Because platelet P2Y12 reactivity analyzed using VerifyNow is easy and fast (less than 5 min), we argue that in patients with acute ischemic stroke, a baseline platelet P2Y12 reactivity assessment before clopidogrel prescription is feasible and should be considered to identify those at risk of HCPR.

Subjects with a poor clopidogrel responsive score of 2 and 3 had HRs of 5.4 and 17.4 for developing HCPR, respectively (Fig. 2). CHANCE-2, a recently published clinical trial, compared another P2Y12 inhibitor (ticagrelor) with clopidogrel in stroke patients who had symptoms within 24 h and were at risk of HCPR^28^. Rapid *CYP2C19* LoF genotyping was achieved with an average turnaround time of 85 min in this clinical trial. They found that among Chinese patients with minor ischemic stroke or TIA who were carriers of *CYP2C19* LoF alleles, the risk of recurrent stroke was modestly lower with ticagrelor than with clopidogrel^28^. The present study showed that the etiology of clopidogrel resistance was multifactorial and heterogeneous. In future clinical studies or trials, our score integrating the *CYP2C19* LoF genotypes with the other HCPR-related clinical factors may also have the ability, potentially with more precision, to detect a clinical benefit with tailored antiplatelet therapy in patients with acute stroke.

The present study had limitations. The major limitation was the limited sample size and statistical power, for which we were unable to evaluate the interactions between HCPR-related factors and validate the sensitivity and specificity of the developed risk score. In addition, the comprehensive statistical validation might not be able to be conducted owing to the limited sample size and a lack of experimental arm. Similarly, the small number of events owing to the limited sample size might have contributed to our failure to detect a clinical event-predictive value of our developed risk score. Additionally, although we found significant predictive factors for HCPR in the present study, a small proportion of HCPR patients (5.8%) did not have any of these factors. Furthermore, a considerable proportion of patients with a poor clopidogrel response score of 3 (33.3%) still showed a normal platelet response to clopidogrel. These results indicate that other undetected genetic or clinical factors and their potential interactions are involved in the pathophysiology of HCPR. Further studies with more genetic and molecular candidates are needed to investigate the underlying mechanisms. Potential genetic-targets would include the single nucleotide polymorphisms of other drug-metabolizing enzymes (CYP2C9, CYP3A4, CYP2B6 and CES 1), transporters (ATP-binding cassette subfamily B member 1) and action receptors (P2Y12), other drug-metabolizing enzymes (CYP2C9, CYP3A4, CYP2B6, and CES1), transporters (ATP-binding cassette subfamily B member 1), and action receptors (P2Y12)^29–31^. Lastly, a limitation, but also an advantage, is that our study population comprised only Taiwanese, which might have provided a risk score specific for the Asian population. Therefore, the present results may not be applicable to other ethnic populations.

## Conclusions

**Figure 3:**
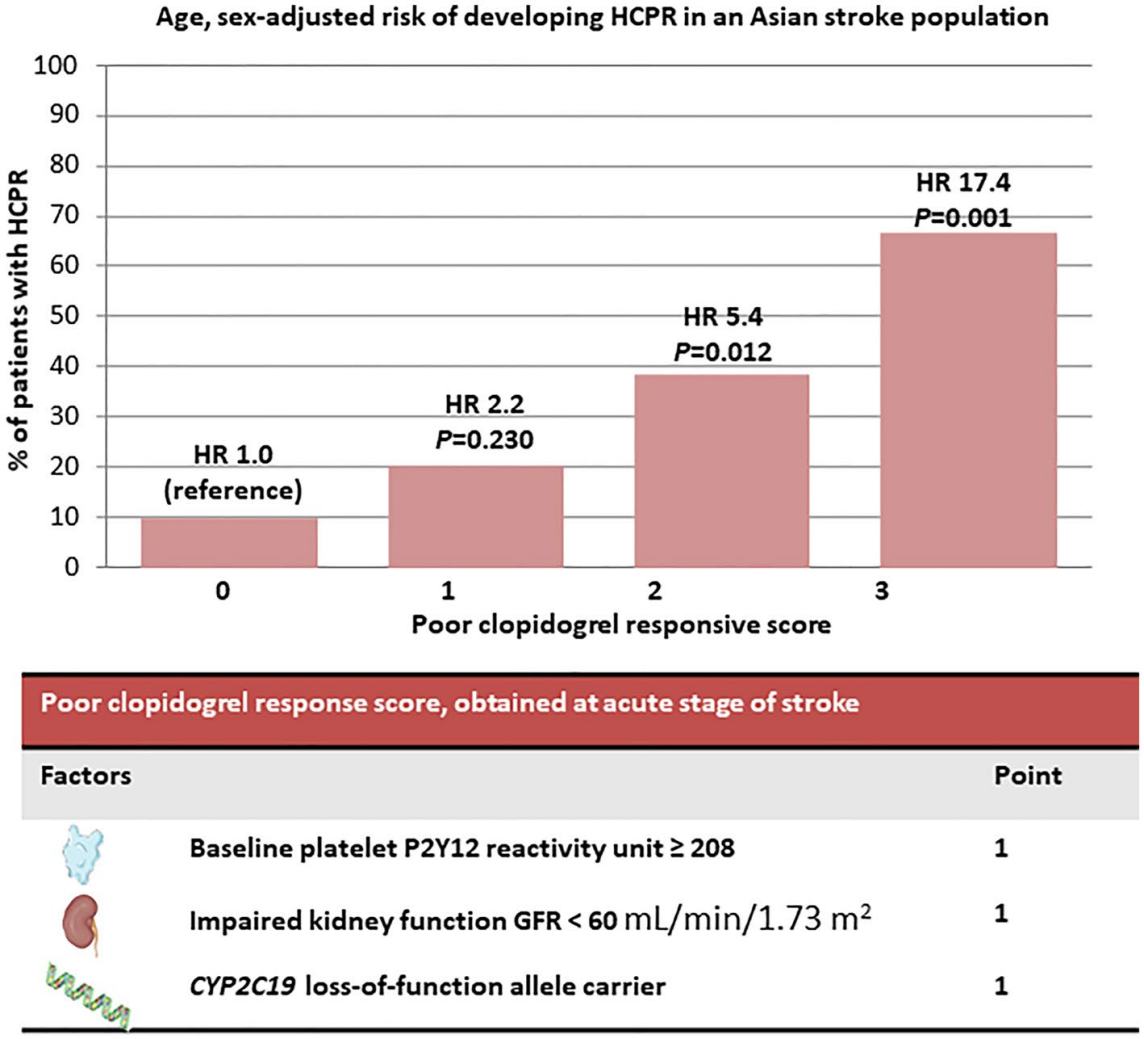
Summary of the study: a poor clopidogrel response score obtained at acute stage of stroke.

The present study emphasized the critical role of HCPR in patients with ischemic stroke. At the acute stage of ischemic stroke, a considerable number of patients had HCPR (27.4%), and HCPR was significantly associated with short-term recurrent events. We also developed a risk score significantly predicting HCPR with three items: high baseline platelet P2Y12 reactivity (PRU value of ≥ 208), impaired kidney function (estimated glomerular filtration rate of < 60 mL/min), and *CYP2C19* LoF allele carrier (Fig. 3). These items could be evaluated soon at the acute stage of stroke before the short-term recurrence events, and provide information to identify patients who are at risk of clopidogrel resistance before antiplatelet agent prescription.

## Data Availability

The data supporting the findings of this study are available from the corresponding author upon reasonable request.
